# Comprehensive Evaluation of the Anti‐Inflammatory Activity of Ling‐Zhi‐8 (LZ‐8) In Vitro and a Rheumatoid Arthritis Animal Model

**DOI:** 10.1002/fsn3.71825

**Published:** 2026-04-29

**Authors:** Shao‐Wen Hung, Po‐Jung Tsai, Cheng‐Yao Yang, Lu‐Te Chuang

**Affiliations:** ^1^ Division of Sustainable Agriculture, Agricultural Facilities and Environment Research Center Agricultural Technology Research Institute Hsinchu City Taiwan; ^2^ Department of Human Development and Family Studies National Taiwan Normal University Taipei City Taiwan; ^3^ Program of Nutritional Science, School of Life Science National Taiwan Normal University Taipei City Taiwan; ^4^ Graduate Institute of Veterinary Pathobiology National Chung Hsing University Taichung City Taiwan; ^5^ Department of Biotechnology and Pharmaceutical Technology Yuanpei University of Medical Technology Hsinchu City Taiwan

**Keywords:** immune regulation, inflammation, LZ‐8, rat, rheumatoid arthritis

## Abstract

Rheumatoid arthritis (RA) is an inflammatory disease caused by an autoimmune reaction. Only immunomodulators or related medicines can decrease RA progression. Ling‐Zhi‐8 (LZ‐8) is a small‐molecule protein in Ganoderma lucidum that inhibits inflammation. This study aimed to investigate the anti‐inflammatory function and immunomodulatory activity of LZ‐8 in RA. Rat RA animal and cell models were used to examine the anti‐inflammatory efficacy of LZ‐8 in inhibiting arthritis. Experimental results indicated that LZ‐8 inhibited the production of interleukin (IL)‐6 and the over‐expression of cyclooxygenase‐2 in lipopolysaccharide‐stimulated macrophages. This suppressive effect may be associated with the potential inhibitory effect of LZ‐8 on the MyD88‐mediated Jun N‐terminal kinase‐mitogen‐activated protein kinase signaling pathway. Administration of lower doses (3.125 mg/kg) of LZ‐8 injections for 9 days reduced inflammation and swelling in the soles of the feet in the RA animal model. The pathological lesion of the limb digits of rats in the LZ‐8 lower doses (3.125 mg/kg) group was relatively milder than those of the medium‐ and high‐dose groups. Comparing the histological lesions, the LZ‐8 high‐dose group showed reduced synovial hyperplasia, and all three doses of LZ‐8 mitigated damage to the articular cartilage. The reduced spleen weight and the distribution of CD4^+^IL‐4^+^, CD4^+^IL‐17^+^, CD4^+^IFN‐γ^+^, and CD4^+^CD25^+^FOXP3^+^ T‐cell immune populations were observed. In conclusion, this study demonstrates that LZ‐8 attenuates inflammatory and immunological responses in a CFA (complete Freund's adjuvant)‐ induced arthritis model, supporting its potential as an immunomodulatory agent for arthritis‐associated inflammatory conditions.

## Graphical abstract

LZ‐8 reduced IL‐6 and COX‐2 in LPS‐stimulated macrophages, partly by inhibiting MyD88‐mediated JNK‐MAPK signaling. LZ‐8 reduced inflammation and swelling in the ankle and palm of the feet in the animal model. High‐dose LZ‐8 reduced synovial hyperplasia, and all doses protected articular cartilage. LZ‐8 reduced spleen weight and altered CD4^+^ T‐cell subset distribution. LZ‐8 significantly attenuates CFA‐induced inflammation, in part by modulating immune cells and suppressing inflammatory responses.

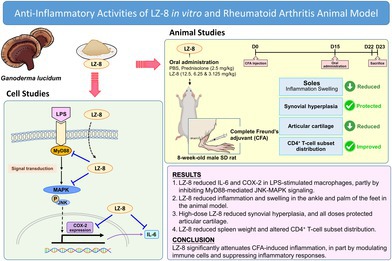

**Abbreviations**
CFA: complete Freund's adjuvant
CIA: collagen‐induced arthritis
COX‐2: cyclooxygenase‐2
DCs: dendritic cells
DMEM: Dulbecco's Modified Eagle Medium
ELISA: enzyme‐linked immunosorbent assay
ERK: extracellular signal‐regulated kinase
FBS: fetal bovine serum
FDR: false discovery rate
H&E: hematoxylin and eosin
IACUC: Institutional Animal Care and Use Committee
IFN: interferon
IL: interleukin
IRAK: IL‐1 receptor‐associated kinase
JNK: Jun N‐terminal kinase
LPS: lipopolysaccharide
LZ‐8: Ling‐Zhi‐8
MAPK: mitogen‐activated protein kinase
MMP‐13: matrix metalloproteinase‐13
NF‐κB: nuclear factor kappa‐light‐chain‐enhancer of activated B cells
PBS: phosphate‐buffered saline
PVDF: polyvinylidene difluoride
RA: rheumatoid arthritis
rLZ‐8: recombinant Ling‐Zhi‐8
STAT3: signal transducer and activator of transcription 3
TLR: toll‐like receptor
TNF: tumor necrosis factor
TRAF6: TNF receptor‐associated factor 6

## 1 Introduction

Rheumatoid arthritis (RA) is one of the most common chronic inflammatory diseases, often presenting with autoantibodies and associated with joint damage and increased mortality in the most severe patients (Smolen et al. 2016). When autoantibodies remain on the synovial membrane of the joints, the leukocytes clear the autoimmune complexes through an inflammatory response. This persistent inflammatory response is also the cause of joint damage in RA (McInnes and Schett 2011). The inflammatory response of RA synovitis originates from innate immune cells (dendritic cells [DCs], monocytes) and adaptive immune cells (helper T cells‐1, helper T cells‐17, and B cells). These cells and immune complexes trigger a strong inflammatory response in the joint area, leading to the destruction of joint tissue (Liu et al. 2013; McInnes and Schett 2011; Smolen et al. 2007). Ling‐Zhi‐8 (LZ‐8) is a commonly used immunomodulatory protein isolated from *Ganoderma lucidum* (Reishi mushroom). It was first discovered and characterized in 1989 (Kino et al. 1989). LZ‐8 consists of 110 amino acid residues, most of which form a homodimer polypeptide with a molecular weight of approximately 24 kDa (Huang et al. 2014; Yang et al. 2014). Each monomer of LZ‐8 consists of an N‐terminal α‐helical domain and a C‐terminal β‐sheet‐rich domain, connected by a flexible linker region (Huang et al. 2014).

LZ‐8 exerts complex effects on the immune system. In an asthmatic mouse model, LZ‐8 regulates activation of signal transducer and activator of transcription 3 (STAT3), thereby inhibiting Th17 development and reducing inflammation (Liu et al. 2020). The dry extract of *Ganoderma lucidum* modulates T lymphocyte function, increases the expression of interleukin (IL)‐10 and tumor growth factor‐β, and promotes the proportion of Th2 cells, suggesting that LZ‐8 may play a role in regulating the Th1/Th2 balance and anti‐inflammatory response (Iser‐Bem et al. 2024). In addition to mediating inflammatory responses, LZ‐8 modulates immune responses in macrophages (Huang et al. 2014). LZ‐8 activates multiple components of the mitogen‐activated protein kinase (MAPK) signaling pathway, including extracellular signal‐regulated kinase (ERK), Jun N‐terminal kinase (JNK), and p38, thereby promoting the maturation and activation of DCs, which showed higher levels of co‐stimulatory molecules (e.g., CD80, CD83, CD86) and cytokines (e.g., tumor necrosis factor [TNF]‐α, IL‐6, IL‐2, interferon [IFN]‐γ), thereby modulating the effects of the immune system (Lin et al. 2009).

LZ‐8 has anti‐inflammatory effects in cells but also in animal experiments. LZ‐8‐treated nonobese diabetic mice exhibited no cumulative incidence of diabetes, whereas untreated mice showed an incidence of up to 70%. LZ‐8 treatment also alters T‐cell subpopulations, which indicates LZ‐8 has immunomodulatory effects (Kino et al. 1990). In a mouse model of asthma, recombinant LZ‐8 (rLZ‐8) significantly reduced lung and inflammatory cell infiltration. It modulates the immune response by decreasing IL‐17 and increasing IL‐10 levels, and by shifting toward regulatory T cells. These effects, modulated by rLZ‐8, are associated with the suppression of the STAT3 and nuclear factor kappa‐light‐chain‐enhancer of activated B cells (NF‐κB) signaling pathways (Liu et al. 2020). In animal studies, LZ‐8 modulates immune responses, particularly by enhancing the function of regulatory T cells, which play a crucial role in controlling excessive inflammation (Xiao et al. 2020).

Lipopolysaccharide (LPS) is a structural component of the outer membrane of Gram‐negative bacteria. It is used in experimental models to mimic innate immune activation and inflammatory responses associated with RA (Akira and Takeda 2004). LPS primarily signals through Toll‐like receptor 4 (TLR4) on immune cells, activating the NF‐κB pathway to promote the expression of pro‐inflammatory cytokines, including IL‐1β, IL‐6, and TNF‐α (Liu et al. 2017). These cytokines are also important mediators of synovial inflammation in RA (Akira and Takeda 2004; Liu et al. 2017).

In animal studies, LPS administration—either systemically or intra‐articularly—has been shown to intensify collagen‐induced arthritis (CIA), a common RA model, by increasing leukocyte infiltration, synovial thickening, and bone erosion.

In this study, we hypothesized that LZ‐8, an anti‐inflammatory modulator, may effectively alleviate inflammatory responses in cells and in a CFA (complete Freund's adjuvant)‐induced mono‐arthritis rat model. We evaluated the anti‐inflammatory effects of LZ‐8 on immune cells and its anti‐inflammatory properties in this adjuvant‐induced arthritis model to investigate its ability to alleviate arthritis‐like joint inflammation, which is distinct from systemic models such as CIA.

## 2 Materials and Methods

### 2.1 Cells

The murine RAW264.7 macrophage cells and human chondrosarcoma SW1353 cells were purchased from the Bioresource Collection and Research Center, Food Industry Research and Development Institute (FIRDI; Hsinchu, Taiwan). The cell culture protocols followed the instructions provided for the certificates. Briefly, RAW264.7 cells were cultured in Dulbecco's Modified Eagle Medium (DMEM) media with 10% (v/v) fetal bovine serum (FBS) and maintained in an incubator with 5% CO_2_ at 37°C (Huang et al. 2014). Human chondrosarcoma SW1353 cells were cultured in Leibovitz's L‐15 medium with 2 mM L‐glutamine with 10% (v/v) FBS in a 37°C incubator.

### 2.2 Viability and Inflammatory Responses to the Cell

Pure recombinant LZ‐8 was purchased from Yeastern Biotech (Taipei, Taiwan) and dissolved in sterile water (10 μg/mL) (Lin et al. 2011). Lipopolysaccharide (LPS) prepared from *Escherichia coli* O26: B6 and 3‐(4,5‐dimethylthiazol‐2‐yl)‐2,5‐diphenyl‐tetrazolium bromide were purchased from Sigma Chemical Co. (St. Louis, MO, USA). Mouse IL‐6 enzyme‐linked immunosorbent assay (ELISA) kits were purchased from eBioscience (Cat. # 88‐7064‐88, Thermo Fisher Scientific Inc., Waltham, MA, USA).

To evaluate the effects of LZ‐8 on cell viability, RAW264.7 cells (1 × 10^5^ cells/mL) were cultured in a 96‐well culture plate; incubated in a 37°C incubator for 2 h; replaced with fresh DMEM with 1, 5, 10 and 25 μg/mL of LZ‐8; and incubated at 37°C for 24 h. Human chondrosarcoma SW1353 cells were treated, as previously described, but using fresh Leibovitz's L‐15 medium supplemented with 0.25, 0.5, 1.0, and 2.5 μg/mL of LZ‐8. Both experiments were performed in triplicate, and each sample was performed with four technical replicates. The MTT assay was used in this study to assess cell proliferation, and trypan blue dye exclusion was also performed. Images were captured using a Nikon Eclipse TS100 microscope (Nikon, Tokyo, Japan).

To examine the effect of LZ‐8 on cellular inflammatory mediators, RAW264.7 cells were treated with 0, 5, or 10 μg/mL of LZ‐8 for 24 h, followed by 24 h of LPS treatment (0.1 μg/mL). The cell‐free supernatants were examined for IL‐6 concentration using commercial kits according to the manufacturer's instructions. The RAW264.7 cells were collected and lysed using lysis buffer (Bio‐Rad Laboratories Inc., Hercules, CA, USA). Twenty micrograms of protein from cell lysates were separated by 10% (w/v) sodium dodecyl sulfate‐polyacrylamide gel electrophoresis and transferred onto polyvinylidene difluoride (PVDF) membranes (Millipore, Burlington, MA, USA). The PVDF membranes were hybridized with an anti‐cyclooxygenase‐2 (COX‐2) antibody (1:1000 dilution; Cat. # 610204, BD Biosciences, Franklin Lakes, NJ, USA), anti‐matrix metalloproteinase‐13 (MMP‐13) antibody (1:1000 dilution; Cat. # ab315267, Abcam, Cambridge, UK), or anti‐MyD88 antibody (1:1000 dilution; Cat. # mAb #50010, Cell Signaling, Beverly, MA, USA) and then reacted with alkaline phosphatase‐conjugated anti‐rat or anti‐rabbit IgG (1:5000 dilution; Cat. # AP136A or AP132A, Sigma, St. Louis, MO, USA). The target COX‐2 protein was visualized using the 5‐bromo‐4‐chloro‐3‐indolyl phosphate/nitro blue tetrazolium substrate (Sigma, St. Louis, MO, USA). Protein signals were measured and quantified by densitometry (Image Lab, Bio‐Rad).

### 2.3 Time Course and MAPK Protein Phosphorylation in SW1353 Cells

Human chondrosarcoma SW1353 cells were seeded in 6‐well plates at a density of 2.25 × 10^5^ cells/well. The cells were cultured for 24 h, and the medium was removed. Next, 1 mL of fresh culture medium containing LZ‐8 was added. After 24‐h incubation, the cell media were removed again, and 1 mL of serum‐free culture medium containing 0.5 ng/mL IL‐1β was added to each well. The cells were cultured for 0, 15, 30, 45, and 60 min. The cells were lysed in lysis buffer to collect cellular proteins, which were analyzed using western blotting.

### 2.4 Animal Study and Experimental Design

Thirty‐three 8‐week‐old male Sprague–Dawley rats (200–250 g) were obtained from BioLASCO Taiwan Co. Ltd. (Yilan, Taiwan). The rats were housed in a 12‐h light/dark cycle at 23°C–25°C and 70%–75% relative humidity. A regular laboratory diet (PANLAB SL, Barcelona, Spain) and freshwater were provided *ad libitum*. All procedures were approved by the Institutional Animal Care and Use Committee (IACUC) of Agricultural Technology Research Institutes (approval No. 105131) and the IACUC of Yuanpei University of Medical Technology (approval No. 10504).

Briefly, the rats were divided into six groups (A–F). Group A (three rats) served as a blank control group, receiving neither induction nor administration; this minimal sample size was chosen to adhere to the 3Rs (Replacement, Reduction, Refinement) principles for animal welfare while establishing a normal baseline. Groups B, C, D, E, and F, with six rats per group, justified by a priori power calculations to achieve > 80% statistical power for primary inflammatory endpoints, were induced to have inflammatory arthritis with 0.1 mL CFA by intra‐articular injection of the right ankle on animal study day 0 (D0). On D14–D22, the rats in groups B and C were administered via intra‐articular (IA) injection with 0.1 mL once a day of sterile water and prednisolone (2.5 mg/kg, dissolved in sterile water; TaFong Pharmaceutical Co. LTD, DOI DOH Manufacturing No. 010886) as the vehicle and positive controls, respectively. The rats in groups D, E, and F were administered via IA injection with 0.1 mL of LZ‐8 (dissolved in sterile water as the vehicle) at 12.5, 6.25, and 3.125 mg/kg, respectively, once a day. The rationale for selecting these doses was based on our preliminary pilot studies and previous literature regarding the effective range of in vivo LZ‐8 administration. We set 12.5 mg/kg as the maximum dose to evaluate potential dose‐dependent therapeutic efficacy and to monitor for any high‐dose immune‐stimulatory effects or toxicity. All the rats were sacrificed on D23.

On D6 and D13–D23, the body weight, paw thickness, ankle circumference, and arthritis scores of the right hind limb of all rats were assessed. Pathological changes in the right ankle joints of the rats were evaluated by staining the pathological slides with hematoxylin and eosin (H&E) and toluidine blue. The spleen weight and splenocyte populations were also assessed.

The IACUC approved animal experiments of the Animal Technology Laboratories, Agricultural Technology Research Institute (Affidavit of Approval of Animal Use Protocol, IACUC No. 105131), and animal care was performed in compliance with the “Guidelines for the Care and Use of Laboratory Animals” of the Council of Agriculture, Taiwan.

### 2.5 The Spleen Weight and Splenocyte Populations of Rats

After the rats were euthanized, their spleens were immediately weighed, moved to a laminar flow hood on ice, cut into pieces, and placed in a sterile disposable syringe. The spleens were ground by squeezing them through the syringe tip with the plunger and then passing them through a nylon mesh. The splenocytes were suspended in phosphate‐buffered saline (PBS).

The splenocytes were suspended in PBS. For intracellular cytokine staining (IL‐4, IL‐17, and IFN‐γ), the isolated splenocytes (1 × 10^6^ cells/mL) were pre‐stimulated with phorbol 12‐myristate 13‐acetate (PMA, 50 ng/mL) and ionomycin (1 μg/mL) in the presence of a protein transport inhibitor (Brefeldin A, 10 μg/mL) for 4–6 h at 37°C. After stimulation and washing, cells were first surface‐stained with the fluorescein isothiocyanate‐conjugated anti‐rat CD4 antibody (clone OX35, Cat. # 11–0040‐81, eBioscience, Thermo Fisher Scientific Inc., Waltham, MA, USA), PE‐conjugated anti‐rat CD25 antibody (clone OX39, Cat. # MA1‐80772, Invitrogen, Carlsbad, CA, USA), in the dark at 4°C. Subsequently, the cells were fixed and permeabilized using a commercial intracellular fixation and Permeabilization buffer set (eBioscience, Thermo Fisher Scientific Inc., Waltham, MA, USA) according to the manufacturer's protocol. The permeabilized cells were then stained with intracellular antibodies, including PE‐conjugated anti‐rat IL‐4 antibody (clone OX81, Cat. # 555082, BD Bioscience), PerCP Cyanine5.5‐conjugated anti‐rat IL‐17 antibody (clone eBio17B7, Cat. # 45‐7177‐82, eBioscience, USA), PE‐conjugated anti‐rat IFN‐γ antibody (clone DB‐1, Cat. # 50‐170‐040, Biolegend, San Diego, CA, USA), and PerCP Cyanine5.5‐conjugated anti‐rat FOXP3 (clone FJK‐16S, Cat. # 45‐5773‐82, eBioscience). Appropriate isotype controls and fluorescence‐minus‐one controls were prepared in parallel to establish precise gating boundaries and distinguish positive staining from background fluorescence. The gating strategy involved initial identification of the live lymphocyte population based on forward scatter (FSC) and side scatter (SSC) characteristics, followed by gating on the CD4^+^ population to determine the frequencies of specific intracellular cytokine‐ or transcription factor‐expressing subsets. Staining sets were purchased. All labeled cell populations were differentiated using flow cytometry (BD FACSCalibur, Becton, Dickinson, and Company, Franklin Lakes, NJ, USA) with appropriately calibrated instrument settings and the data were processed using the WinMDI (version 2.9) software.

### 2.6 Statistical Analysis

For the in vivo study, the sample sizes (*n* = 6 per treatment group) were calculated and justified to ensure at least 80% statistical power at a 5% significance level (*α* = 0.05) to detect meaningful differences in primary clinical and histological scores. Statistical comparisons among multiple groups were initially performed using a one‐way analysis of variance (ANOVA) followed by an appropriate post hoc test.

To account for the increased risk of Type I errors due to testing multiple simultaneous endpoints—specifically spleen weight, levels of IL‐6, MMP‐13, COX‐2, and the frequencies of the four T‐cell subsets (CD4^+^ IL‐4^+^, CD4^+^ IL‐17^+^, CD4^+^ IFN‐γ^+^, and CD4^+^ CD25^+^ FOXP3^+^)—the Benjamini‐Hochberg procedure was applied to control the false discovery rate (FDR) across multiple endpoints. An FDR‐adjusted *p* < 0.05 was considered statistically significant.

## 3 Results

### 3.1 Effects of LZ‐8 on Cell Viability and Inflammatory Responses

**FIGURE 1:**
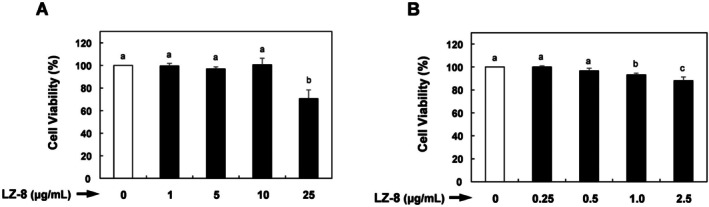
Effects of LZ‐8 on the cell viability of RAW264.7 macrophages (A) and chondrosarcoma SW1353 (B). RAW264.7 and SW1353 cells were treated with LZ‐8 for 24 h. Data are presented as means ± standard deviations (*n* = 4, technical replicates) and were analyzed by one‐way analysis of variance. Different letters indicate significant differences between the two groups.

To determine whether LZ‐8 was cytotoxic to murine RAW264.7 macrophages or human chondrosarcoma SW1353 cells, the culture medium was supplemented with different concentrations of LZ‐8. There was no cytotoxic effect on cell viability when RAW264.7 cells were incubated with LZ‐8 concentrations < 10 μg/mL (Figure 1A). Similarly, no significant negative effect on SW1353 cells was observed when the LZ‐8 concentration in the medium was < 0.5 μg/mL (Figure 1B).

**FIGURE 2:**
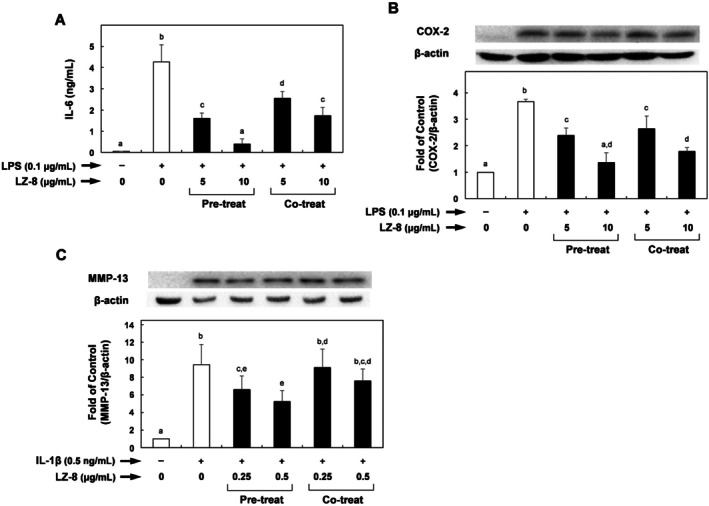
LZ‐8 on LPS‐induced interleukin (IL)‐6 concentration (A) and COX‐2 expression levels (B) of RAW264.7; LZ‐8 on IL‐1β‐induced matrix MMP‐13 concentration (C) of chondrosarcoma SW1353. RAW264.7 cells were treated with LZ‐8 (0.1 μg/mL) or IL‐1β (0.5 ng/mL) for 24 h. IL‐6 concentrations (A) were measured in the supernatants using an enzyme‐linked immunosorbent assay. Amounts of COX‐2 (B) and MMP‐13 protein (C) were quantified using β‐Actin as a loading control and were expressed relative to those controls. Data are presented as mean ± standard deviations from three independent experiments (*n* = 3, biological replicates) and were analyzed by one‐way analysis of variance. *p*‐values were adjusted for multiple comparisons using the Benjamini‐Hochberg false discovery rate (FDR) procedure. Values that do not share the same letter are significantly different at *p* < 0.05.

To examine whether LZ‐8 might affect immune responses by modulating inflammatory mediator production and expression, RAW264.7 cells were pre‐treated with a fixed concentration (5 or 10 μg/mL) of LZ‐8 for 24 h, followed by 24‐h LPS stimulation (0.1 μg/mL). Results of Figure 2A,B show that LPS markedly enhanced IL‐6 synthesis and COX‐2 overexpression of COX‐2 compared with the untreated control. LZ‐8 pre‐treatment significantly suppressed IL‐6 and COX‐2 levels by up to 91% and 63%, respectively (Figure 2A,B). In addition to pre‐incubating macrophages with LZ‐8 before LPS stimulation, we investigated whether LZ‐8 exerted a similar suppressive effect on inflammatory responses when RAW264.7 cells were co‐cultured with LZ‐8 and LPS for 24 h. Our results demonstrated that LZ‐8 significantly reduced LPS‐stimulated IL‐6 production and COX‐2 expression when the concentration of LZ‐8 was > 5 μg/mL (Figure 2A,B).

We determined whether LZ‐8 modulates inflammatory chondrocytes by suppressing MMP‐13 expression in the matrix. Figure 2C shows that the pre‐treatment with LZ‐8 significantly reduced MMP‐13 expression by up to 45% compared with that without pre‐treatment. However, the co‐treatment with LZ‐8 did not have such a suppressive effect.

### 3.2 Effect of LZ‐8 on MyD88 and MAPK Activation

**FIGURE 3:**
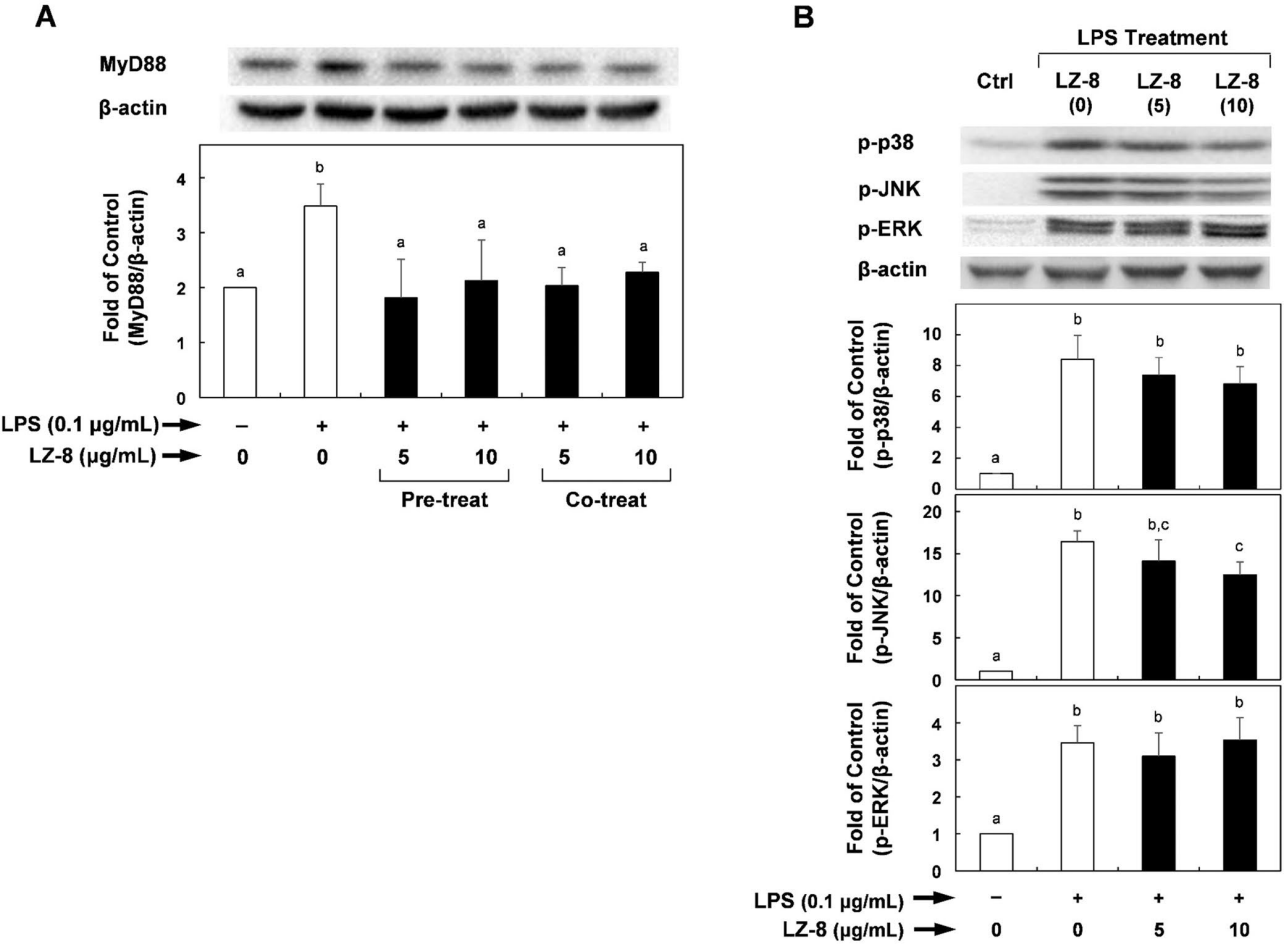
Effects of LZ‐8 on LPS‐induced MyD88 expression levels and LPS‐induced phospho‐MAPK protein levels in RAW264.7 cells. RAW264.7 cells were treated with LZ‐8 for 24 h and then induced with LPS (0.1 μg/mL) for 24 h. MyD88 protein (A) and p‐p38, p‐JNK, and p‐ERK (B) were quantified using β‐actin as a loading control and were expressed relative to that control. Data are presented as means ± standard deviations from three separate experiments (*n* = 3, biological replicates) and were analyzed by one‐way analysis of variance. Values that do not share the same letter are significantly different at *p* < 0.05.

To determine the mechanisms underlying the suppressive effects of LZ‐8 on the levels of IL‐6 and COX‐2 in macrophages, we examined the effect of LZ‐8 on the expression of MyD88 in RAW264.7 cells. Relative to the negative control, the level of MyD88 increased in response to LPS stimulation (Figure 3A). However, pre‐incubation or co‐incubation of macrophages with different doses of LZ‐8 significantly suppressed the LPS‐stimulated MyD88 expression by up to 48% and 42%, respectively.

LPS stimulates inflammatory responses by activating TLR/MyD88‐mediated MAPK signaling (Park et al. 2009). We further investigated whether the inactivation of MAPK by LZ‐8 plays a central role in modulating pro‐inflammatory mediators. Figure 3B shows that phosphorylated p38‐, JNK‐, and ERK–MAPK were upregulated in response to LPS. Phosphorylation of JNK in LPS‐stimulated RAW264.7 cells pre‐incubated with LZ‐8 was significantly suppressed when the LZ‐8 concentration was 10 μg/mL (Figure 3B). However, LZ‐8 either slightly attenuated or had no effect on the activation of p38 and ERK.

**FIGURE 4:**
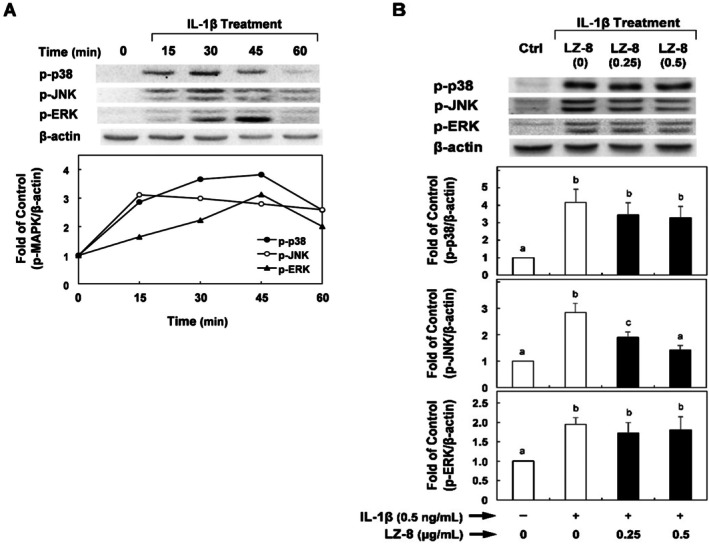
Effects of LZ‐8 on interleukin (IL)‐1β‐induced MyD88 expression levels and IL‐1β‐induced phospho‐MAPK protein levels in SW1353 cells. (A) Time course study of IL‐1β‐induced phospho‐MAPK protein levels in SW1353 cells was evaluated over 15–60 min. (B) SW1353 cells were treated with LZ‐8 for 24 h, and then incubated with IL‐1β (0.5 ng/mL) for 30 min. Amounts of p‐p38, p‐JNK, and p‐ERK were quantified using β‐Actin as a loading control and were expressed relative to those controls. Data are presented as means ± standard deviations from three separate experiments (*n* = 3, biological replicates) and were analyzed by one‐way analysis of variance. Values that do not share the same letter are significantly different at *p* < 0.05.

Furthermore, Li et al. recently reported that activation of MAPK signaling was strongly associated with MMP overexpression in chondrosarcoma cells (Zeng et al. 2017). Thus, we examined whether LZ‐8 can reduce MAPK‐mediated inflammatory responses. Figure 4A shows a time course analysis of IL‐1β‐stimulated MAPK activation in SW1353 cells. Based on these results, we selected 30 min as the optimal time for MAPK activation. The results in Figure 4B show that the levels of phosphorylated p38, JNK, and ERK in SW1353 cells increased in response to IL‐1β‐induced stimulation compared with the negative control. Pre‐treatment of cells with LZ‐8 significantly reduced phosphorylated JNK levels by up to 50% compared with the IL‐1β‐stimulated control. The levels of phosphorylated p‐38 and ERK were only slightly reduced; however, these effects were not statistically significant.

### 3.3 The Clinical Observation of Rats in Groups

**FIGURE 5:**
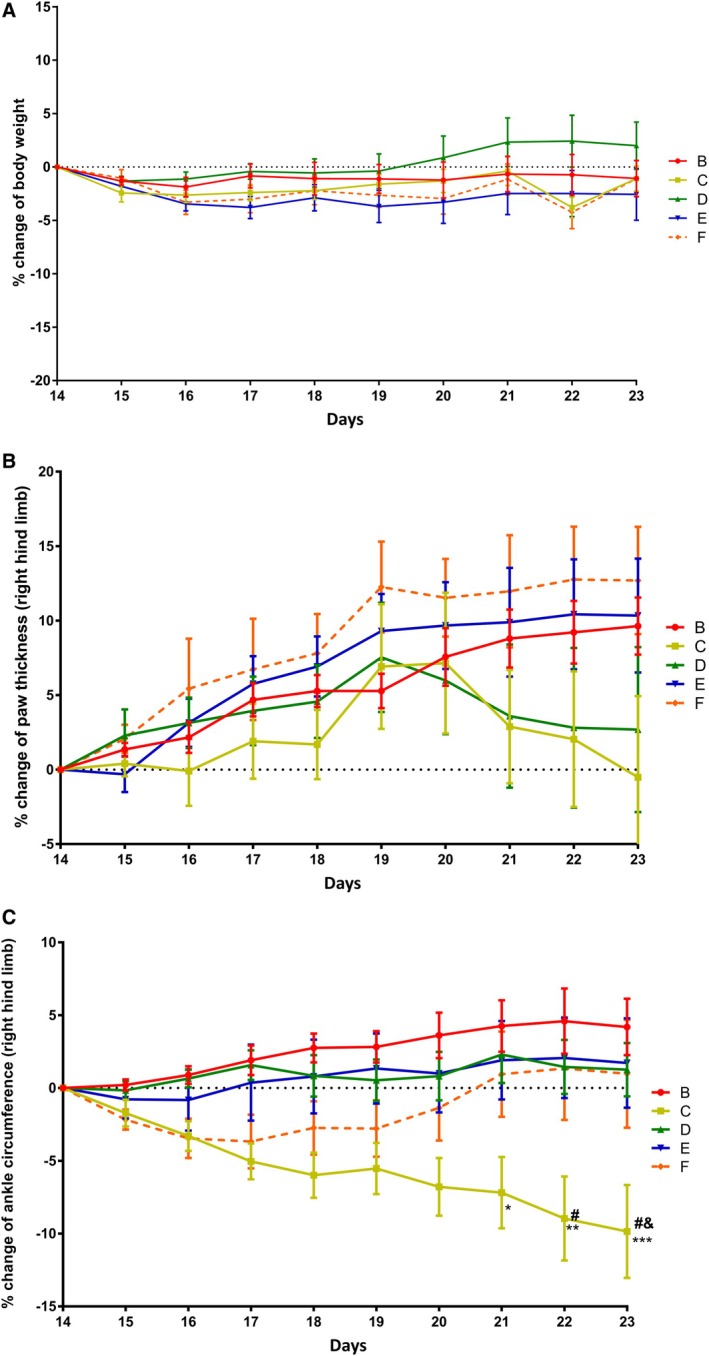
The changes of body weight, right hind paw thickness, and the circumference of the right hind ankle of rats. (A) Changes in body weight (%) of rats after treatment. The changes in body weight were compared with the weight on the rat's first treatment day (day 14). (B) The change (%) in the right hind paw thickness of rats. The right hind paw thicknesses of the treatment groups (B, C, D, E, F) were compared daily with those of the blank control group (group A). (C) The change (%) in the circumference of the right hind ankle of rats. The circumferences of the right hind ankles of the treatment groups (B, C, D, E, and F) were compared daily with those of the blank control group (group A). (*/**/***p < 0.05/0.01/0.001, group C vs. group B; ^#^ *p* < 0.05, group C vs. group E; ^&^ *p* < 0.05, group C vs. group D).

The rats were weighed on D6 and D13–D23. Body weights were compared to those on the first day of treatment (D14). The weights of rats in all treated groups (groups B‐F) slightly decreased within 3 days (D15–D17) after treatment, and then returned slowly to normal. During the treatment schedule (D14–D23), no significant difference (*p* > 0.05) in body weight was observed among the groups (Figure 5A).

### 3.4 The Inflammation Evaluation in the Right Paw and the Right Hind Ankle of Rats

Paw thickness and ankle circumference of the right hind limb were evaluated for inflammation. The daily change rate (%) in the thickness of the rat's right hind paw and the circumference of the right hind ankle was based on the change in thickness and circumference compared with those on the first day of treatment (D14). The change (%) in the right hind paw thickness of rats in each treatment group (group B–F) during the treatment period (D14–D23) was not significantly different. The positive control group (group C) and the 12.5 mg/kg of 100% pure LZ‐8 (group D) showed reduced paw thickness (Figure 5B). Another inflammatory parameter, the daily change (%) in the circumference of the right hind ankle of the rats in each test group, was compared on D14 to D23 during the treatment period. Swelling of the right hind ankle significantly subsided in the positive control group treated with prednisolone (Group C). The positive control group (group C) had a significantly reduced ankle circumference of the right hind limb (Figure 5C).

### 3.5 Histological Assessment of the Right Hind Ankle of Rats

**FIGURE 6:**
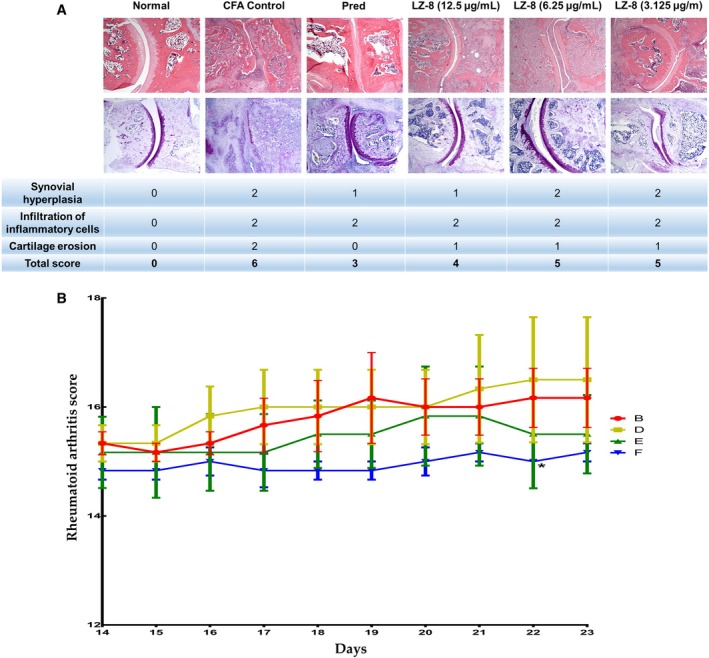
The pathological changes in the right ankle joints of rats were assessed using rheumatoid arthritis pathological scores. (A) The pathological lesion of the longitudinal section of the tibiotalar joint with hematoxylin and eosin staining (upper) and toluidine blue staining (lower) in the right hind limb. Original magnification, 40×. The scores for synovial hyperplasia, inflammatory cell infiltration, and cartilage erosion were listed in the table. (B) Daily assessment of total rheumatoid arthritis scores in each group of rats. The rheumatoid arthritis pathological scores of the treated groups (groups D, E, F) were compared with those of the vehicle control group (group B) daily (*p < 0.05).

The RA pathological scores were assessed according to the method described (Wang et al. 2005) as follows: score 0, normal; score 1, pathological area < 1/2; and score 2, pathological area ≥ 1/2 (Figure 6A). Pathological changes, including synovial hyperplasia, inflammatory cell infiltration, and cartilage erosion, were assessed. The total score from the three lesion assessments for each rat was used to compare pathological changes among the rats in each group. The RA pathological scores of groups E (LZ‐8 6.25 mg/kg) and F (LZ‐8 3.125 mg/kg) were lower than those of the vehicle group (group B) during the treatment period (D14–D23), and the scores in group F significantly subsided on D22. The LZ‐8 3.125 mg/kg group (group F) rats showed the lowest RA pathological scores during the treatment period the pathological damage to the tibiotalar joints in the right hind limb (Figure 6B).

### 3.6 The Spleen Weight and Splenocyte Populations of Rats

**FIGURE 7:**
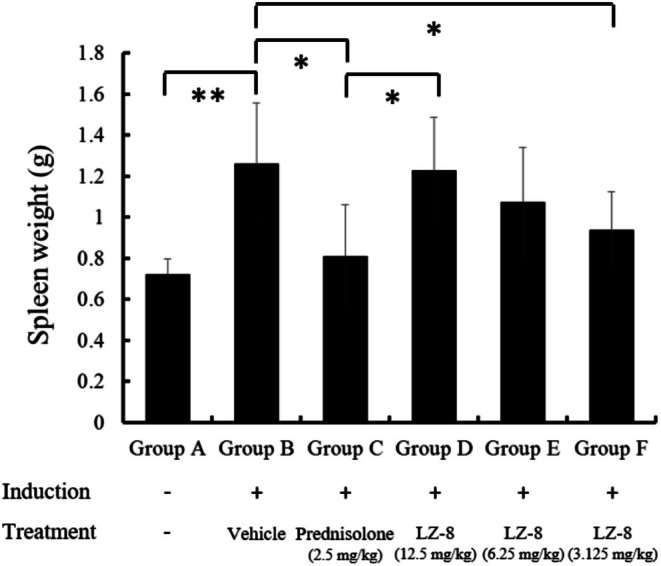
The spleen weights of the rats in groups. Data are presented as means ± standard deviations (*n* = 3–6). Statistical significance was evaluated using one‐way ANOVA. *p*‐values were adjusted for multiple comparisons using the Benjamini‐Hochberg false discovery rate (FDR) procedure. *p < 0.05 and **p < 0.01 represent significant differences compared with the vehicle control group (Group B).

An inflammatory reaction in the spleen increased the body weight, indicating a systemic inflammatory response. The spleen weights were positively correlated with the treatment dosage in groups D, E, and F (Figure 7). The spleen weights of the blank control group (group A), positive control group (group C), and 3.125 mg/kg LZ‐8 group (group F) were significantly lower than those of group B (vehicle control). In contrast, those of group F did not differ significantly from those of groups A and C. The spleen weight of the rats in group D (LZ‐8, 12.5 mg/kg) was significantly higher than that of the rats in group C (positive control, prednisolone, 2.5 mg/kg). There was no significant difference in spleen weight between rats in group E (6.25 mg/kg group) and those in groups B and C, but the degree of inflammation differed between these two treatment groups. This indicates that group C rats recovered to approaching regular spleen weights after prednisolone treatment from D14 to D23. Rats treated with 3.125 mg/kg (group F) from D14 to D23 showed a similar reduction in inflammation and approached normal spleen weights as rats in group C.

### 3.7 The Splenocyte Populations of Rats

**FIGURE 8:**
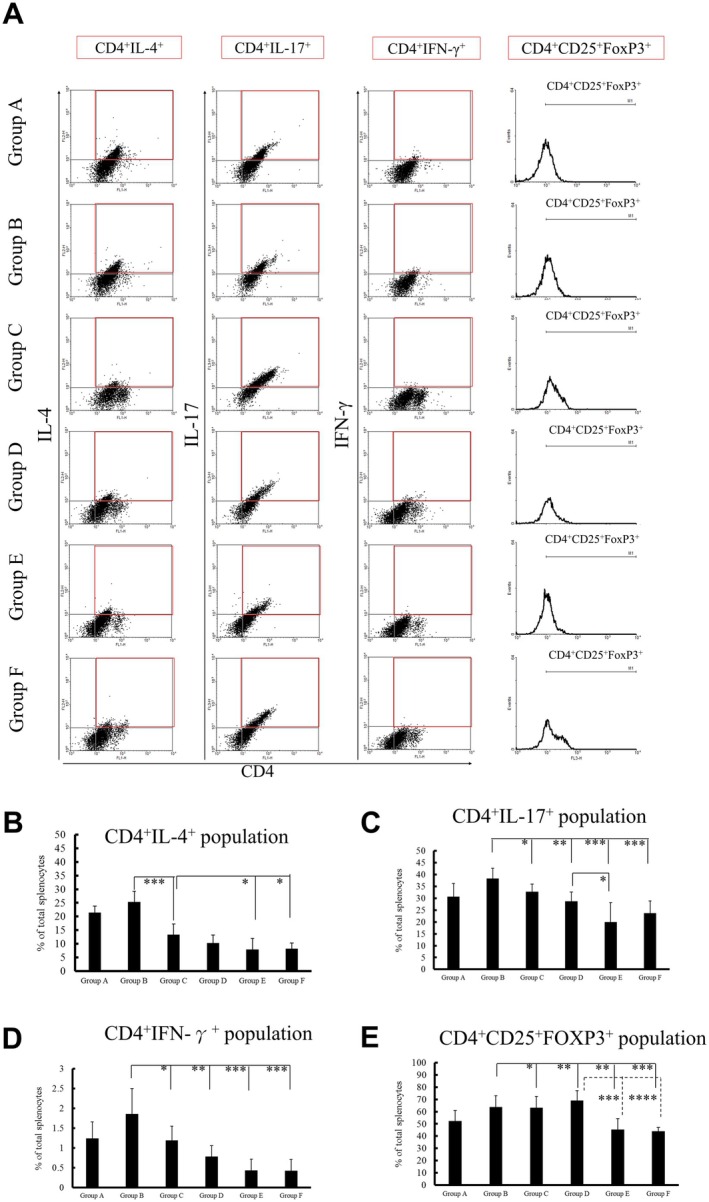
Gating hierarchy of the splenocyte population of rats. (A) The histogram was used to identify the CD4^+^, CD25^+^, and FoxP3^+^ populations. Within the CD4^+^ populations, the IL‐4^+^, IL‐17^+^, and IFN‐γ^+^ cells were in the upper‐right quadrant (Three columns to the left, red frame). The histograms were gated on viable CD4^+^CD25^+^FoxP3^+^ cells (rightmost column). (B) Percentage of CD4^+^IL‐4^+^ cell population in spleen cells of rats in each group. (C) Percentage of CD4^+^IL‐17^+^ cell population in spleen cells of rats in each group. (D) Percentage of CD4^+^ IFN‐γ^+^ cell population in spleen cells of rats in each group. (E) Percentage of CD4^+^CD25^+^FoxP3^+^ cell population in spleen cells of rats in each group. Six rats were in each group except group A (three rats; *n* = 3–6). Statistical significance was evaluated using one‐way ANOVA. *p*‐values were adjusted for multiple simultaneous endpoints using the Benjamini‐Hochberg false discovery rate (FDR) procedure. *p < 0.05, **p < 0.01, and ***p < 0.001 represent significant differences compared with the vehicle control group (Group B).

Antibodies against specific cell markers on the splenocytes determined the splenocyte populations. The CD4^+^, CD25^+^, and FoxP3^+^ populations in rat splenocytes were tested and identified (Figure 8A). The percentage of CD4^+^IL‐4^+^ cells population in the spleen cells of group B rats was significantly higher than that in group C rats and higher than that in groups E and F rats (Figure 8B). The percentages of CD4^+^IL‐17^+^ and CD4^+^ IFN‐γ^+^ cell populations in the spleen cells of rats in group B was higher than those in groups C, D, E, and F (Figure 8C,D). The percentage of CD4^+^CD25^+^FoxP3^+^ cells in the spleen cells of group D rats was higher than that in groups B, C, E, and F (Figure 8E).

## 4 Discussion

The primary finding of this study was that LZ‐8 exerted an anti‐rheumatic effect in a CFA‐induced rat model of RA by suppressing the production and expression of pro‐inflammatory mediators, which was associated with the downregulation of JNK‐MAPK signaling in murine macrophages and human chondrocytes, thereby achieving anti‐inflammatory effects.

In RAW264.7, we demonstrated that LZ‐8 significantly suppressed LPS‐stimulated pro‐inflammatory IL‐6 production and downregulated COX‐2 expression. These results align with the anti‐inflammatory properties of LZ‐8 reported in previous studies using LPS‐stimulated murine RAW264.7 macrophages, BV‐2 microglia, and carbon tetrachloride‐induced rat models (Huang et al. 2014). These results are similar to those of previous studies on the anti‐inflammatory effects of RA (H. Liu et al. 2020).

When examining the potential mechanisms underlying the immunoregulatory effects of LZ‐8, our results showed that LZ‐8 treatment was associated with reduced MyD88 protein abundance and decreased JNK‐MAPK phosphorylation. MyD88 acts as a key adaptor protein for TLR signaling and plays a crucial role in the development of LPS‐stimulated inflammation (Deguine and Barton 2014). For example, after LPS binds to TLR4, MyD88 spontaneously recruits and activates a series of downstream kinases, including IL‐1 receptor‐associated kinase‐4 (IRAK‐4), IRAK‐1, and TNF receptor‐associated factor 6 (TRAF6), subsequently leading to the activation of MAPK and NF‐κB cellular signaling, which triggers the overexpression and production of a wide range of inflammatory mediators (J. Chen et al. 2020). The results of this study suggest that LZ‐8 may have a similar effect on joint inflammation.

LZ‐8 regulates the MAPK signaling pathway, including p38, JNK, and ERK, thereby affecting immune cell functions and potentially exerting therapeutic effects on inflammatory diseases, such as arthritis (Lin et al. 2009). The PI3K signaling pathway is notably involved in RA pathogenesis (Gonzalez‐Lopez de Turiso et al. 2012). Our results also confirmed that LZ‐8 exerts an anti‐inflammatory effect via the MAPK pathway, reducing arthritis‐associated lesions, as evidenced by pathological changes. While these results suggest that LZ‐8 has immunomodulatory properties, any claims regarding its direct application for the development of RA‐related immuno‐modulatory nutrients or supplements must be tempered. Comprehensive preclinical studies, including detailed efficacy, toxicology, and immunogenicity data, are still necessary before establishing its true therapeutic implications.

In chronic, systemic inflammatory diseases such as RA, many immune cells, including Th1, Th2, Tregs, natural killer cells, and even B cells, are involved in inflammatory responses (Weyand and Goronzy 2021). Th1 cells release inflammatory cytokines (such as IFN‐γ, TNF‐α, and IL‐1β) to promote inflammation in areas where it is induced. However, IL‐4 produced by Th2 cells can attenuate the anti‐inflammatory cytokine response in RA (Ruterbusch et al. 2020). In this study, flow cytometry was used to monitor Th1, Th2, and Treg cells in the spleen of LZ‐8‐treated rats. We found that Th1 (CD4^+^IFN‐γ^+^) and Th2 (CD4^+^IL‐4^+^) cells in the spleens of rats treated with prednisolone (group C) and those treated with LZ‐8 (groups D‐F) were significantly lower than in the induced rat (group B). Tregs (CD4^+^CD25^+^FOXP3^+^) in the induced rats and treated rats did not differ. The profiles of Th1, Th2, and Treg cells in splenocytes were similar to those observed in a tolerogenic DC‐treated animal model of collagen‐induced arthritis (Yuan et al. 2024). The repression of inflammation may be associated with the regulation of CD4^+^ T cell balance (Yuan et al. 2024). LZ‐8 activated immune responses by enhancing the expression or production of TNF‐α in lymphocytes and macrophages (Huang et al. 2014). This anti‐inflammatory FIP suppressed inflammatory responses by reducing TLR4‐mediated NF‐κB signaling in LPS‐activated BV‐2 microglia (Chen et al. 2017). Similarly, FIPs from *Trametes versicolor* or *Hericium erinaceus* have been reported to inhibit LPS‐activated macrophages and suppress the production of inflammatory cytokines (Diling et al. 2017).

Inhibition of osteoclast differentiation and promotion of apoptosis were observed in rLZ‐8‐treated rats, thereby reducing bone resorption. This effect may be achieved by regulating the RANK‐TRAF6‐JNK signaling pathway, which has the potential to prevent the bone destruction commonly observed in RA (Ruan et al. 2019). In a rat model of glucocorticoid‐induced osteoporosis, rLZ‐8‐treated rats showed a bone loss‐prevention effect on trabecular morphology, as observed by H&E staining. This study showed that the LZ‐8 anti‐inflammatory effect is involved in bone and related tissues (Yang et al. 2020). In our study, pathological examination revealed that rats treated with LZ‐8 showed reduced pathological damage to the induced tibiotalar joints of the right hind limb, as indicated by a smaller pathological score. The rats also exhibited slight inflammation, characterized by small changes in paw thickness and circumference of the right hind ankle, indicating that LZ‐8 has an anti‐inflammatory effect on the joint damage caused by RA.

We summarized our findings demonstrating that LZ‐8 attenuated inflammatory and immunological responses in a CFA‐induced mono‐arthritis rat model, accompanied by anti‐inflammatory effects observed in RAW264.7 macrophages. LZ‐8 suppressed the pro‐inflammatory response and significantly reduced MAPK activation, which was associated with the amelioration of arthritis‐associated joint pathology, as evidenced by histopathological changes. In addition, LZ‐8 suppresses the production of inflammatory cytokines by lymphocytes and macrophages, potentially attenuating bone resorption and joint destruction commonly observed in inflammatory arthritis.

Regarding the dose–response relationship, the in vivo results revealed an intriguing phenomenon where the lowest dose of LZ‐8 (3.125 mg/kg) outperformed higher doses (12.5 and 6.25 mg/kg) in improving clinical and histological scores. A positive correlation was observed between the LZ‐8 dose and spleen weight, with the high‐dose group (12.5 mg/kg) showing significantly heavier spleens. This inverse dose‐dependent efficacy and dose‐dependent splenomegaly suggest that LZ‐8 possesses complex dual immunomodulatory properties. In previous studies (Lin et al. 2009; Zeng et al. 2017), LZ‐8 exerts anti‐inflammatory effects but can also activate the MAPK signaling pathway, promoting dendritic cell maturation and activation, upregulating co‐stimulatory molecules and pro‐inflammatory cytokines such as TNF‐α and IL‐6. We hypothesize that at higher doses, the immunostimulatory properties of LZ‐8 predominate, leading to systemic immune hyper‐activation reflected by increased spleen weight and potential off‐target toxicity, ultimately counteracting its localized anti‐inflammatory benefits in the joints. Contrariwise, the lower dose (3.125 mg/kg) appears to achieve an optimal therapeutic window, effectively downregulating inflammatory signaling without triggering excessive, counterproductive immune stimulation.

A notable limitation of this study is that the precise molecular targets and signaling networks modulated by LZ‐8 remain incompletely defined. Specifically, because no loss‐of‐function (e.g., MyD88 knockdown/knockout) or pharmacologic (e.g., SP600125 for JNK) experiments were performed, a direct causal link between LZ‐8 and the MyD88‐JNK signaling pathway cannot be definitively established. The detailed mechanisms underlying T‐cell regulation and long‐term immune responses require further investigation.

Further studies are required to delineate the specific receptors, intracellular pathways, and immune cell interactions involved in the immunomodulatory actions of LZ‐8. For example, transcriptomic profiling of T cells and macrophages treated with LZ‐8 can help identify differentially expressed genes and pathways associated with immune modulation. In addition, investigation of signaling pathways downstream of pattern recognition receptors and T‐cell receptors, including TLR‐ and MyD88‐ associated signaling, may provide further mechanistic insights. Longitudinal in vivo studies are essential for evaluating sustained immunomodulatory effects, which inform the durability and therapeutic potential of LZ‐8. Because our study utilized a CFA‐induced mono‐arthritis model rather than a systemic CIA model, the direct translation of these findings to human RA requires caution. Therefore, extensive future studies focusing on comprehensive efficacy, off‐target toxicology, and in vivo immunogenicity will be necessary to evaluate the durability and scope of LZ‐8‐mediated immunomodulatory effects in inflammatory arthritis models.

## 5 Conclusion

In conclusion, LZ‐8 significantly reduces the CFA‐induced inflammatory responses, at least in part, by regulating immune cells and suppressing inflammation. However, the precise mechanisms of immune cell interaction require further investigation.

## Author Contributions

**Shao‐Wen Hung:** methodology, investigation, resources, data curation, writing – original draft, visualization, funding acquisition. **Cheng‐Yao Yang:** conceptualization, methodology, validation, data curation, writing – review and editing, project administration, funding acquisition. **Po‐Jung Tsai:** visualization, formal analysis, investigation, writing – original draft, validation. **Lu‐Te Chuang:** conceptualization, methodology, validation, resources, writing – review and editing, supervision, project administration, funding acquisition.

## Funding

This research was funded by the Ministry of Science and Technology, grant number NSC 106‐2320‐B‐264‐001‐, and by the Fund from National Chung Hsing University (grant number 108GP04376), for which we are grateful.

## Ethics Statement

This study was conducted in accordance with an animal use protocol approved by the Institutional Animal Care and Use Committee (IACUC) of Agricultural Technology Research Institutes (approval No. 105131) and IACUC of Yuanpei University of Medical Technology (approval No. 10504).

## Conflicts of Interest

The authors declare no conflicts of interest.

## Data Availability

The data that support the findings of this study are available from the corresponding author upon reasonable request.
